# De novo Y1460C missense variant in Na_V_1.1 impedes the pore region and results in epileptic encephalopathy

**DOI:** 10.1038/s41598-022-22208-x

**Published:** 2022-10-13

**Authors:** Quentin Plumereau, Aya Ebdalla, Hugo Poulin, Juan Pablo Appendino, Morris H. Scantlebury, Ping Yee Billie Au, Mohamed Chahine

**Affiliations:** 1 CERVO Brain Research Center, 2601, de la Canardière, Quebec City, QC G1J 2G3 Canada; 2 Department of Medical Genetics, Alberta Children’s Hospital Research Institute, Cumming School of Medicine, University of Calgary, Calgary, AB Canada; 3 Neurology Section, Department of Pediatrics, Alberta Children’s Hospital, Cumming School of Medicine, University of Calgary, Calgary, AB Canada; 4 Department of Medicine, Faculty of Medicine, Université Laval, Quebec City, QC Canada

**Keywords:** Biophysics, Molecular biophysics, Diseases, Neurological disorders, Epilepsy

## Abstract

Epilepsy is a common neurological disorder characterized by recurrent unprovoked seizures. *SCN1A* encodes Na_V_1.1, a neuronal voltage-gated Na^+^ channel that is highly expressed throughout the central nervous system. Na_V_1.1 is localized within the axon initial segment where it plays a critical role in the initiation and propagation of action potentials and neuronal firing, predominantly in γ-amino-butyric-acid (GABA)ergic neurons of the hippocampus. The objective of this study was to characterize a de novo missense variant of uncertain significance in the *SCN1A* gene of a proband presented with febrile status epilepticus characterized by generalized tonic clonic movements associated with ictal emesis and an abnormal breathing pattern. Screening a gene panel revealed a heterozygous missense variant of uncertain significance in the *SCN1A* gene, designated c.4379A>G, p.(Tyr1460Cys). The Na_V_1.1 wild-type (WT) and mutant channel reproduced in vivo and were transfected in HEK 293 cells. Na^+^ currents were recorded using the whole-cell configuration of the patch-clamp technique. This Na_V_1.1 variant (Tyr1460Cys) failed to express functional Na^+^ currents when expressed in HEK293 cells, most probably due to a pore defect of the channel given that the cell surface expression of the channel was normal. Currents generated after co-transfection with functional WT channels exhibited biophysical properties comparable to those of WT channels, which was mainly due to the functional WT channels at the cell surface. The Na_V_1.1 variant failed to express functional Na^+^ currents, most probably due to pore impairment and exhibited a well-established loss of function mechanism. The present study highlights the added-value of functional testing for understanding the pathophysiology and potential treatment decisions for patients with undiagnosed developmental epileptic encephalopathy.

## Introduction

*SCN1A* is one of the most rigorously studied sodium channel genes in epilepsy, with hundreds of genetic variants identified^1,2^. The spectrum of seizure disorders associated with *SCN1A* range from mild to intermediate presentations such as isolated febrile seizure or genetic epilepsy with febrile seizure plus. The most severe and often life-limiting seizure disorders associated with *SCN1A* mutations include epileptic encephalopathies (EE) such as Dravet syndrome (DS, also known as severe myoclonic epilepsy in infancy), intractable childhood epilepsy with generalized tonic–clonic seizures (ICE-GTC), epilepsy of infancy with migrating focal seizures (EIMFS), and myoclonic astatic epilepsy (MAE)^3,4^. *SCN1A* can also be associated with non-epileptic phenotypes such as familial hemiplegic migraine^3,4^. *SCN1A* encodes the α subunit of voltage-gated sodium channels and is widely expressed in neurons of the hippocampus, brainstem, cortex, caudate, substantia nigra, and caudal regions of the brain^2^. Specifically, *SCN1A*, which is located in the axon initial segment (AIS), plays a critical role in the initiation and propagation of action potentials (APs), predominantly in γ-aminobutyric acid (GABA)ergic neurons^5^. Many *SCN1A* pathogenic variants result in protein truncation, leading to channels with complete loss of function (LoF), Missense substitutions have also been reported that can lead to either gain of function (GoF) or LoF^2,6^. Identifying the underlying genetic etiology of epilepsy is of paramount importance as this will help optimize patient outcomes by allowing patient-centered therapies, improved understanding of prognosis, and a more precise understanding of recurrence risk for families^7^.

Access to clinical genetic testing has become more routine in the last decade for many patients with epilepsy, and high-throughput sequencing has markedly improved the diagnostic yield of epilepsy disorders^7,8^. However, confirmation of a genetic diagnosis is frequently hampered by difficulties in interpreting variants of uncertain significance. Although in silico predictors, allele frequency, and de novo inheritance variants can assist with variant classification, there are still limitations using these parameters, particularly for interpreting missense variants. Functional testing can be an invaluable tool for making a definitive diagnosis in addition for potentially assisting in outcome prediction and therapy selection^6^.

We report here a case of a de novo missense variant of uncertain significance in the *SCN1A* gene. Functional testing using patch clamp techniques and cell surface expression analyses indicated, most probably, an impaired pore function that made it possible to reclassify this variant as probably pathogenic.

## Methods

### Cell cultures

Human embryonic kidney 293 (HEK 293) cells were used to express WT and mutant Na_V_1.1/Y1406C sodium channels. The cells were grown in high-glucose Dulbecco’s modified Eagle’s medium supplemented with 10% fetal bovine serum and 1% streptomycin at 37 °C in a 5% CO_2_ atmosphere. The human Na^+^ channel β1-subunit and enhanced Green Fluorescence Protein (eGFP) were inserted in the pIRES bicistronic vector in the form of β1-pIRES-eGFP. The cells were transfected with the plasmid cloning DNA3.1 vector (pCDNA3.1) containing either WT Na_V_1.1 complementary DNA (1 μg) or the Na_V_1.1/Y1460C mutant with the pIRES2/EGFP vector containing β1 subunit complementary DNA (1 μg) and an empty pCDNA3.1 vector in 10-cm cell culture dishes using the calcium phosphate method as previously reported^9^.

### Cell surface biotinylation and western blotting

Biotinylation experiments were carried out using a modification of a previously published method^10^. Briefly, proteins were isolated from HEK293 cells transfected with either WT or mutant Na_V_1.1 channels grown in 100-mm dishes until they reached 80–90% confluence. The cells were washed three times in ice-cold Dulbecco's phosphate-buffered saline (D-PBS) and were then incubated for 2 h at 4 °C with 1 mg of EZ-Link™ Sulfo-NHS-SS-Biotin (Thermo Fisher Scientific, Waltham, MA, USA) in ice-cold D-PSB (pH 8). The reactions were stopped by washing the cells three times for 5 min with 5 mg/mL of BSA (Proliant Biologicals, Ankeny, IA, USA) and 50 mM glycine (MilliporeSigma, MO, USA) in ice-cold D-PBS fallowed by three washes with ice-cold D-PBS. Proteins were extracted in ice-cold lysis buffer (10 mM Tris–HCl, pH 7.4, 150 mM NaCl, 1 mM EDTA, 0.5% sodium deoxycholate, and 1% NP-40) containing cOmplete™ ULTRA (MilliporeSigma) protease inhibitor cocktail. Biotinylated proteins were isolated overnight at 4 °C from 800 µg of total proteins on 25 µL of settled High Capacity NeutrAvidin™ Agarose resin (Thermo Fisher Scientific) in 40 volumes of lysis buffer. The resin was washed 3 times in 40 volumes of ice-cold lysis buffer, and 3 times for 10 min in room temperature lysis buffer under vertical axis agitation. It was then transferred to a spin column for centrifugal elution for 30 min at 37 °C with 35 µL of elution buffer (lysis buffer 0.1X, 35 mM NaCl, 25 mM tris(2-carboxyethyl)phosphine (TCEP)) (Thermo Fisher Scientific). Laemmli sample buffer was added to the biotinylated samples before loading them on an SDS-PAGE gel.

Proteins were extracted from HEK293 cells transfected with Na_V_1.1/WT or Na_V_1.1/Y1460C in lysis buffer (10 mM Tris–HCl, pH 7.4, 150 mM NaCl, 1 mM EDTA, 0.5% sodium deoxycholate, and 1% NP-40) containing cOmplete™ ULTRA (MilliporeSigma). Cleared lysates were dosed using BCA Protein Assay kits (Thermo Fisher Scientific). Total protein (10 µg) was slightly denatured in Laemmli sample buffer (BioRad) containing 25 mM TCEP (Thermo Fisher Scientific) for 30 min at 37 °C, resolved on 4–15% gradient TGX stain-free polyacrylamide gels (BioRad), and blotted onto 0.2 µm polyvinylidene difluoride (PVDF) membranes (BioRad). The membranes were blocked and were incubated overnight at 4 °C with rabbit anti-Na_V_1.1 antibody (1:500, Alomone Labs, Israel), rabbit anti-sodium potassium NaKATPase antibody (1:10,000, Abcam, Cambridge, UK), or rabbit anti-glyceraldehyde 3-phosphate dehydrogenase (GAPDH) antibody (1:10,000, Bethyl Laboratories-FORTIS Life Sciences, Waltham, MA, USA). Horseradish peroxidase (HRP)-conjugated anti-rabbit antibody was used as the secondary antibody (Cell Signaling Technology, MA, USA). Proteins were revealed using Clarity Western ECL substrates (BioRad) and were visualized using the ChemiDoc MP system (BioRad).

### Whole-cell patch-clamp Na^+^ current recordings

Na^+^ currents were recorded using low-resistance, fire-polished patch clamp electrodes (≈ 1–1.5 MΩ) made from 8161 Corning borosilicate glass coated with HIPEC (Dow-Corning, Midland, MI, USA) to minimize electrode capacitance. An Axopatch 200 amplifier and pClamp software (Molecular Devices, Sunnyvale, CA, USA) were used to record Na^+^ currents. The series resistance was compensated to 80% to minimize voltage-clamp errors. The cells were allowed to stabilize for 5 min after the whole-cell configuration was established. The membrane potential was held at − 140 mV before the currents were recorded. Sodium currents were filtered at 5 kHz and were digitized at 83.33 kHz. The liquid junction potential was not corrected. All the experiments were performed at room temperature (22 °C).

### Recording solutions

The intracellular solution was composed of 35 mM NaCl, 105 mM cesium fluoride, 10 mM ethylene glycol-bis(β-aminoethyl ether)-*N*,*N*,*N*′,*N*′-tetraacetic acid (EGTA), and 10 mM 4-(2-hydroxyethyl)-1-piperazineethanesulfonic acid (HEPES). The pH was adjusted to 7.3 with 2 M CsOH. The external solution (full Na^+^) used was composed of 150 mM NaCl, 2 mM KCl, 1.5 mM CaCl_2_, 1 mM MgCl_2_, 10 mM glucose, and 10 mM HEPES. The pH was adjusted to 7.4 with 2 M HCl.

### Data analysis

The slope factor (k) and the midpoint (V_1/2_) for activation and inactivation were calculated using standard Boltzmann functions: 1/(1 + exp [(V_1/2activation_ − V)/k_activation_]) for activation and (1 − C)/(1 + exp [(V − V_1/2inactivation_)/k_inactivation_] + C) for inactivation. V is the voltage and C is a constant*.*

### Statistical analysis

Results are expressed as means ± standard error of the mean (SEM). Statistical comparisons were performed using a one-way analysis of variance in GraphPad Prism (La Jolla, CA) for statistical comparisons. Differences were considered significant at *P* < 0.05.

### Ethical publication statement

We confirm that we have read the Journal’s position on issues involved in ethical publication and affirm that this report is consistent with those guidelines.

## Results

### Description of the proband

The proband was a female born at full-term by uncomplicated vaginal delivery with forceps to healthy non-consanguineous parents with a non-contributory family history. The proband developed normally until 4 months of age when she was brought to the emergency room with a temperature of 39.1 °C in febrile status epilepticus characterized by generalized tonic clonic movements associated with ictal emesis and abnormal breathing patterns that lasted 75 min. The episode was aborted with phenytoin and phenobarbital. On examination she was macrocephalic with a head circumference of 47 cm (97th percentile). The general and neurological examinations were normal. On neurological examination, she was alert and active. Her cranial nerve and motor development was initially normal, and the parents reported that the proband had learned to roll over by 4 months of age and walk by 10 months of age. Electroencephalogram (EEG), CT head, and MRI brain performed at 4 months of age were normal. Plasma amino acids and urinary organic acids were normal.

In addition to episodes of febrile status epilepticus, the proband exhibited two other seizure types. Six weeks following her initial presentation she presented with a second seizure type consisting of right limb jerks and stiffening of the face. These events occurred with or without fever. At 12 months of age, she presented with a third seizure type consisting of impaired awareness, blank stares, and rhythmic eye movements occasionally triggered by fever and flashing lights.

The focal motor seizures were initially responsive to levetiracetam but eventually became refractory. Both the generalized tonic clonic and focal impaired awareness seizures did not respond to treatment. The parents were unwilling to try other medications but agreed to initiate a ketogenic diet. The awake EEG remained refractory to treatment, with noted epileptiform discharges.

At the final follow up at 16 months of age, the parents reported a marked reduction in seizure frequency following the initiation of the ketogenic diet. However, there was expressive speech delay. EEG performed at 16 months of age showed focal epileptiform discharges over the right frontal region and intermittent focal right central and diffuse slowing. The findings indicated a predisposition to focal onset seizures in addition to regional and global cerebral dysfunction.

### Genetic testing

**Figure 1:**
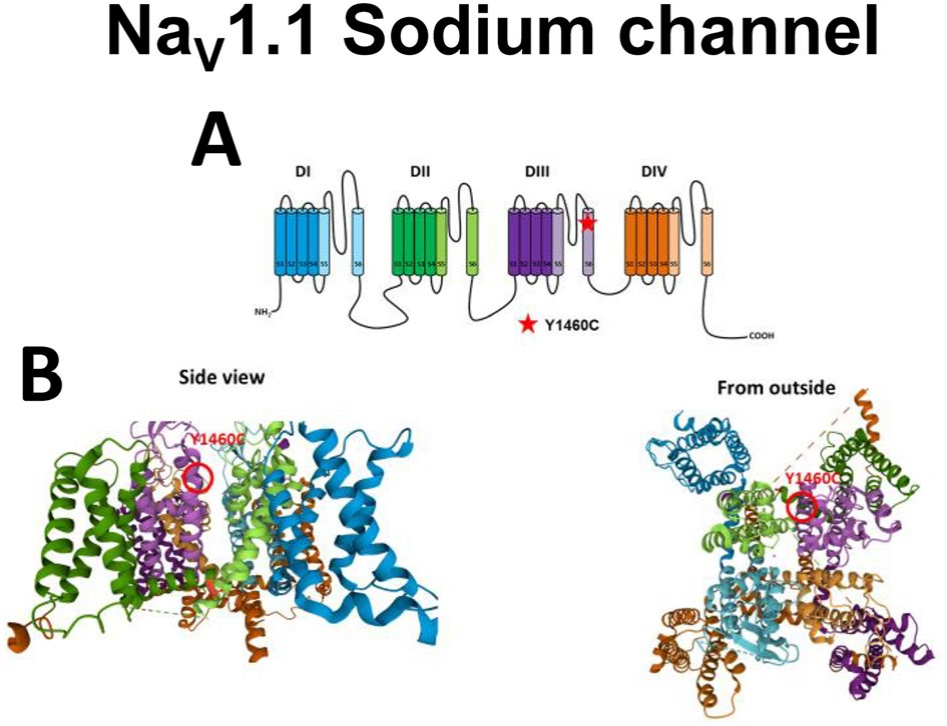
Localization of the Y1460C mutation in the Na_V_1.1 channel. (**A**) Schematic of the secondary structure of the Na_V_1.1 voltage-gated sodium channel. The four homologous domains (DI–DIV), the transmembrane segments (S1–S6), and the extra- and intracellular linkers are shown. The four domains are indicated by different colors while the transmembrane segments forming the pore domain (PD) are indicated by lighter colors. The position of Y1460C is indicated. **(B)** Structural model from Na_V_1.1 Cryo-EM showing the localization of the Y1460C residue with inside and outside views.

Genetic testing was performed by Blueprint Genetics, a clinical diagnostic laboratory. Three hundred seventy-nine genes associated with epilepsy were sequenced as part of the comprehensive epilepsy gene panel. Clinical familial variant testing was also performed by Blueprint Genetics to determine the inheritance of the variant. The gene panel investigation revealed a novel heterozygous missense variant in the *SCN1A* gene, designated c.4379A>G, p.(Tyr1460Cys) (NM_001165963.1), hereafter referred to as Y1460C in the electrophysiology experiments described below. This rare variant is missing from the gnomAD database [accessed April 2022]. Multiple in silico predictors (SIFT, PolyPhen, and Mutation Taster) indicated that the variant was damaging. This Na_V_1.1 mutation was located on the 6th transmembrane segment of domain III, which forms part of the channel pore (Fig. 1). However, this variant has never been described in association with an *SCN1A* seizure disorder. It was inherited from the proband’s asymptomatic father, who was identified as mosaic, with the variant present in 13% of reads (22/167). Y1460C was initially reported as being of uncertain significance by the clinical lab but could also be considered as being likely pathogenic depending on the interpretation of ACMG criteria (the PM1, PM2, PP2, PP3, and mosaicism in the father could be interpreted as de novo)^11^.

The proband was also found to be heterozygous for a de novo variant of uncertain significance in the *KMT2E* gene, c.776C>T, p.(Ala259Val) (NM_182931.2). This gene has been implicated in a neurodevelopmental disorder with varying degrees of intellectual disability that can present with or without epilepsy in macrocephalic individuals. However, this specific variant has not been described in any affected individuals. It is also absent from the gnomAD database. Some in silico predictors indicated that this variant is damaging (PolyPhen, Mutation Taster) whereas others indicated that it may be benign (SIFT). It is thus unclear whether the *SCN1A* variant was causative, or whether the patient’s clinical issues were attributable to the *KMT2E* variant. Confirmation of the pathogenicity of the *SCN1A* variant is important to determine the recurrence risk for the family given the presence of mosaicism in the father and to direct future choices of medication therapy for epilepsy.

### Biophysical and biochemical mutation characterization

**Figure 2:**
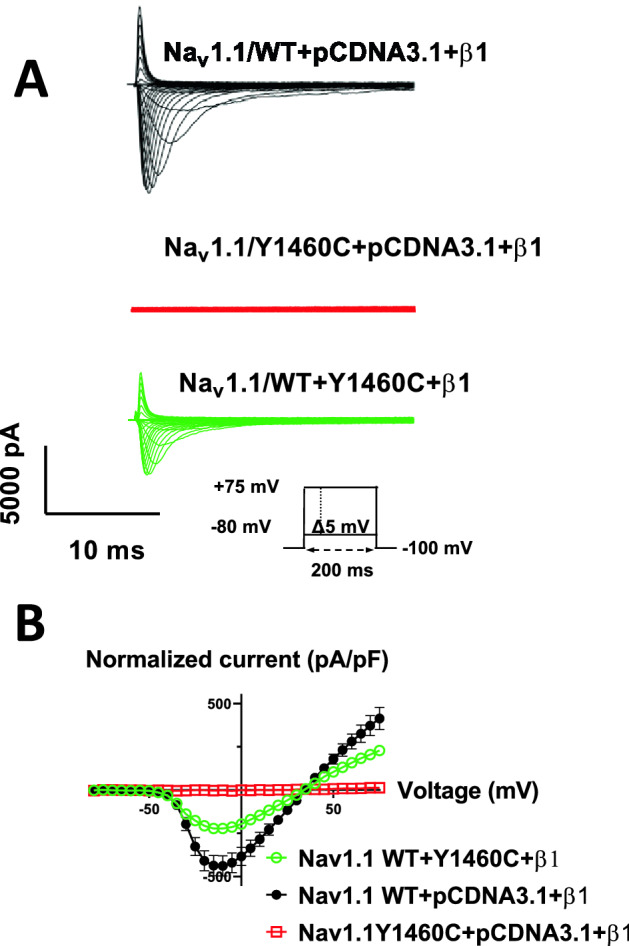
Whole-cell Na^+^ currents recorded from HEK 293 cells. (**A**) Example of raw current traces from Na_V_1.1/WT with Y1460C or empty vector (pCDNA3.1) and Y1460C were obtained using depolarizing pulses from − 80 to + 75 mV in 5-mV increments in a 15 mM Na^+^ solution. (**B**) Analysis of whole-cell Na^+^ currents recorded from HEK 293 cells expressing Na_V_1.1 WT with Y1460C or pCDNA3.1 and Y1460C. Current density–voltage relationship of WT with pCDNA3.1 (Black symbols, n = 13), WT with Y1460C (Red symbols, n = 13), and Na_V_1.1/Y1460C (Green symbols, n = 6), the standard errors are smaller than the symbols. Current amplitudes were normalized to membrane capacitance to obtain the current density (pA/pF).

**Figure 3:**
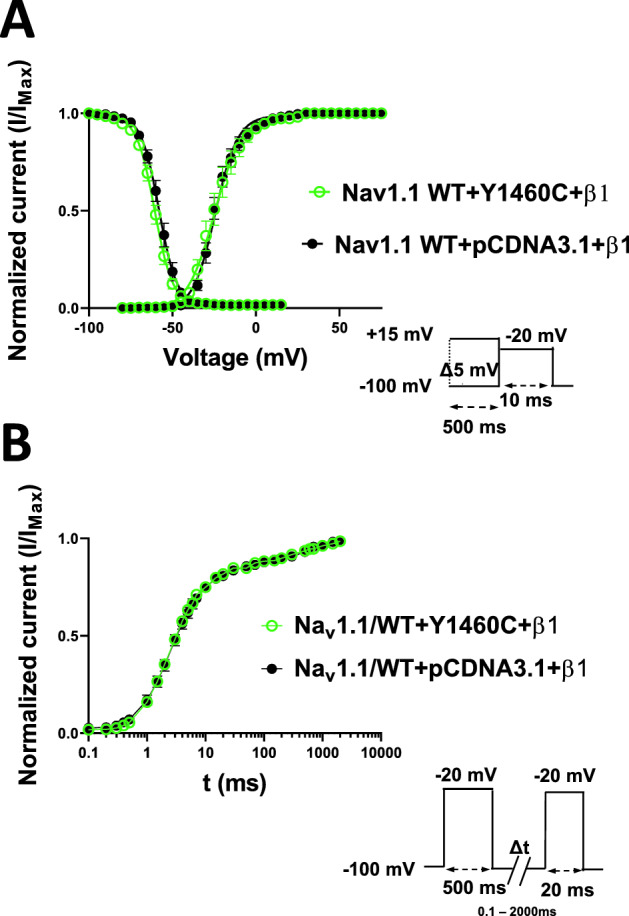
Gating properties of steady-state activation and inactivation and recovery from slow inactivation. (**A**) Voltage-dependence of steady-state activation (left) and inactivation (right) in WT with pCDNA3.1 (activation, n = 11, and inactivation, n = 11) and WT with Y1460C (activation, n = 11, and inactivation, n = 15). The inactivated currents were generated using the protocol described in the insets. and the activated currents were obtained from the I/V recordings. The resulting data were fitted to a standard Boltzmann function. (**B**) Time courses of recovery from slow inactivation in WT with pCDNA3.1 (n = 14) and WT with Y1460C (n = 14).

We compared the biophysical properties of Na_V_1.1/WT to those of Na_V_1.1/Y1460C, both expressed in HEK293 cells. Currents were recorded using sequential depolarizing steps of the cell membrane from − 80 to + 75 mV in 5-mV increments using a 150-mM NaCl external solution. Interestingly, the WT channel was successfully expressed but the mutant channel displayed no currents when it was expressed alone or with the β1 subunit **(**Fig. 2A). We then decided to co-transfect Na_V_1.1/WT with Na_V_1.1/Y1460C and the β1 subunit. As expected, the current density was half that of the Na_V_1.1/WT channel as shown by the current–voltage (I/V) curves (Fig. 2B and Table 1). Each peak of the Na^+^ currents was normalized to the cell capacitance (pA/pF) to construct the I/V curves. We next calculated the G/V curves (steady-state activation) and fitted the data points with a Boltzmann function. No differences were observed with the half-activation potential (V_1/2_) or the slope (kv_act_) (Fig. 3A and Table 1). There was no difference in steady-state inactivation between Na_V_1.1/WT and Na_V_1.1/WT co-transfected with the mutant channel. In addition, there were no significant differences between the half-activation potentials (V_1/2_) or slopes (kv_inc_) (Fig. 3A and Table 1). The recovery from inactivation normalized to the maximum current exhibited no differences between Na_V_1.1/WT and Na_V_1.1/WT co-transfected with the mutant channel (Fig. 3B and Table 1).

**Table 1:** Biophysical parameters of Na_v_1.1/wild type (WT) Na_v_1.1/Y1460C mutant channel.

	Na_v_1.1 WT + pCDNA3.1 + β1	Na_v_1.1 WT + Y1460C + β1
Peak current (pA/pF)	− 438.5 ± 59.0 n = 13	− 222.7 ± 18.9*** n = 13
**Inactivation**		
V_1/2_ (mV)	− 57.9 ± 1.3 n = 11	− 60.6 ± 1.1 n = 15
K (mV)	− 4.6 ± 0.2 n = 11	− 4.8 ± 0.1 n = 15
**Activation**		
V_1/2_ (mV)	− 23.4 ± 1.5 n = 11	− 24.7 ± 2.6 n = 11
K (mV)	6.2 ± 0.6 n = 11	6.2 ± 0.5 n = 11
**Recovery**		
τ fast (ms)	3.7 ± 0.4 n = 14	3.6 ± 0.4 n = 14
τ slow (ms)	311.2 ± 32.6 n = 14	340.1 ± 49.6 n = 14

K, slope factor for activation or inactivation; τ, time constant; V_1/2_, midpoint for activation and inactivation. ***P < 0.001.

### Biotinylation and western blotting

**Figure 4:**
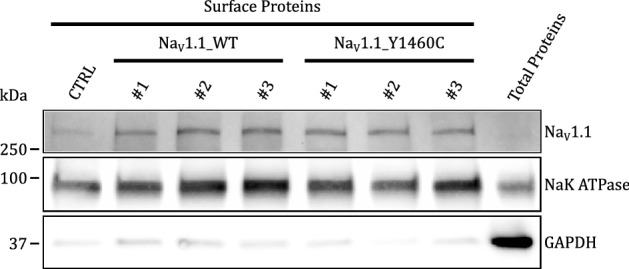
Biotinylation of cell surface proteins and Western blot analysis. Western blot of biotinylated cell surface proteins isolated from HEK293 cells transfected with the Na_V_1.1/WT channel and the Na_V_1.1/Y1460C mutant channel. Cropped images of western blots blots are shown for three different transfections. Original blots are presented in Supplementary Fig. 1 NaKATPase was used as a house keeping gene and GAPDH as a negative control for cytosolic protein contaminants. CTRL represents a non-transfected biotinylated control. Raw Extract represents proteins prior to purification (10 µg).

Given that the mutation was located on the S6 helix that forms the Na_V_1.1 channel pore wall and that fact that no functional currents were generated, we hypothesized that the channel proteins were probably located at the cell surface and that the pore was impaired by the mutation. To test this hypothesis, we carried out biotinylation experiments followed by western blotting to determine whether Na_V_1.1 channel proteins were present at the cell surface. The Western blot revealed the presence of a high molecular weight band (Fig. 4), suggesting the presence of mutant channel proteins at the cell surface. The Western blot also showed that GAPDH, an intracellular protein marker was absent, while the NaKATPase pump, a cell surface protein marker, was present, which indicated that the biotinylation was carried out successfully. Overall, the data suggested that mutant channels are translated and trafficked efficiently to the cell surface but that their activity may be impaired by the mutation, most probably by ion pore impairment.

## Discussion

The proband’s clinical presentation, with onset of focal febrile status epilepticus associated with immunization followed by the emergence of refractory seizures and cognitive development delay, suggests that the proband suffered from epileptic encephalopathy associated with a mutation in *SCN1A*, a condition that is milder than expected in Dravet syndrome. In this study, we confirm that the Y1460C variant of the Na_V_1.1 channel was functionally impaired. The Na_V_1.1/Y1460C variant is located on S6 of DIII, which is part of the Na_V_1.1 pore. The biophysical characterization of the variant revealed a complete loss of current density. As the variant expressed no current at all, other biophysical characterizations were performed by co-transfecting Y1460C with Na_V_1.1/WT. As expected, there were no differences in steady-state activation and inactivation or recovery time. The only effect observed when WT was co-transfected with the variant was a loss of function in peak current density (− 222.7 ± 18.9, Table 1) that was, as expected, 50% of the WT peak current density (− 438.5 ± 59.0, Table 1). This also suggested that there is no dominant negative effect due to the mutation and underscores that the alpha subunit of Nav1.1 channels behaves as a monomer. Biochemical studies using biotinylation and Western blotting showed that Y1460C channel proteins are present at the cell surface, suggesting most probably that the structure of the pore was impaired by the mutation.

There are well established genotype–phenotype correlations for *SCN1A*. Typically, LoF variants which lead to reduced Na_V_1.1 currents, are associated with epilepsy, with the absence of whole cell currents, which most likely leads to DS^6^. In contrast, GoF variants, which exhibit an increase in currents or an impairment of inactivation, are linked to *SCN1A*-related familial hemiplegic migraine^6^.

Many variants identified in *SCN1A*-related EE are missense (including up to 50% missense variants in DS)^1,12^. Unlike protein truncating variants that result in nonsense-mediated decay, LoF missense variants may have different effects depending on whether any Na_V_1.1 protein is produced, whether it is trafficked appropriately, and whether this abnormal protein affects other functions in neurons^13^. Although some missense variants have been functionally assessed by patch clamp to confirm LoF, very few variants have been investigated with respect to how a variant actually results in a reduction in or the absence of cell currents. The underlying cause of reduced currents is of interest, however, as it is possible that improperly trafficked channels may be more amenable to different therapeutic avenues than pore-impaired channels with a normal cell surface expression. The cell surface expression of non-truncating *SCN1A* variants associated with DS, for example, has been shown to be impaired. As such, it may be possible to target them with drugs that boost cell surface expression^14^. However, there is no known drug that can restore the channel function caused by the impairment we have identified. Other therapeutic approaches must thus be used to alleviate the symptoms of this patient and others with similar pore-impairing mutations. This highlights the need and rationale for strategies, such as a ketogenic diet, that act on other pathways, and antisense oligonucleotide therapies, that promote the expression of the functional normal allele of the *SCN1A* gene^15^.

Two variants that affect the same residue (p.(Tyr1460His) (no clinical information available, ClinVar variation ID 871,600) and p.(Tyr1460Asp)(ClinVar variation ID 1,361,552) associated with infantile EE) are listed in ClinVar as variants of uncertain significance. We suspect that, given our findings for Y1460C, these two variants are probably also pathogenic.

For our proband, we suspect that a large part of her clinical picture is attributable to *SCN1A*. However, the impact of the de novo KMT2E variant on the phenotype of the proband is still unclear and may eventually be clarified using novel approaches such as DNA methylation episignature testing given the role of this gene in epigenetic regulation^16^. However, as her father is mosaic for the *SCN1A* variant, there is up to a 50% chance of recurrence risk for future pregnancies. For this patient, confirmation of the pathogenicity of the *SCN1A* variant would have a much higher clinical impact on reproductive planning.

In summary, we report a novel pathogenic *SCN1A* variant, p.Tyr1460Cys, which impacts the Na_V_1.1 pore and causes a complete loss of Na^+^ currents. The present study highlights the value of functional testing for understanding the pathophysiology and potential treatment decisions for patients with undiagnosed developmental epileptic encephalopathy.

## Supplementary Information

Supplementary Information.

## Data Availability

All the data are available upon request to the corresponding author.

## Abbreviations

AP: Action potential
AIS: Axon initial segment
CNS: Central nervous system
DS: Dravet syndrome
EE: Epileptic encephalopathies
EIMFS: Epilepsy of infancy with migrating focal seizures
GABA: γ-amino-butyric-acid
HEK 293: Human embryonic kidney 293
ICE-GTC: Intractable childhood epilepsy with generalized tonic–clonic seizures
MAE: Myoclonic astatic epilepsy
Na_V_1.1: Voltage-gated sodium channel type 1protein
SCN1A: Voltage-gated sodium channel type 1 gene
WT: Wild type

## References

[1] Depienne C, Trouillard O, Saint-Martin C, Gourfinkel-An I, Bouteiller D, Carpentier W (2009). Spectrum of SCN1A gene mutations associated with Dravet syndrome: Analysis of 333 patients. J. Med. Genet..

[2] Oliva M, Berkovic SF, Petrou S (2012). Sodium channels and the neurobiology of epilepsy. Epilepsia.

[3] Miller, I. O., Sotero de Menezes, M. A. SCN1A Seizure disorders. In (eds Adam, M. P., Mirzaa, G. M., Pagon, R. A., Wallace, S. E., Bean, L. J. H., Gripp, K. W. et al.) Seattle. GeneReviews(^®^). Seattle (WA): University of Washington Copyright^©^ 1993–2022, University of Washington, Seattle. GeneReviews is a registered trademark of the University of Washington, Seattle. All rights reserved.1993.

[4] Scheffer IE, Nabbout R (2019). SCN1A-related phenotypes: Epilepsy and beyond. Epilepsia.

[5] Yu FH, Mantegazza M, Westenbroek RE, Robbins CA, Kalume F, Burton KA (2006). Reduced sodium current in GABAergic interneurons in a mouse model of severe myoclonic epilepsy in infancy. Nat. Neurosci..

[6] Brunklaus A, Schorge S, Smith AD, Ghanty I, Stewart K, Gardiner S (2020). SCN1A variants from bench to bedside-improved clinical prediction from functional characterization. Hum. Mutat..

[7] Demos M, Guella I, DeGuzman C, McKenzie MB, Buerki SE, Evans DM (2019). Diagnostic yield and treatment impact of targeted exome sequencing in early-onset epilepsy. Front. Neurol..

[8] Costain G, Cordeiro D, Matviychuk D, Mercimek-Andrews S (2019). Clinical application of targeted next-generation sequencing panels and whole exome sequencing in childhood epilepsy. Neuroscience.

[9] Chahine M, Deschênes I, Chen LQ, Kallen RG (1996). Electrophysiological characteristics of cloned skeletal and cardiac muscle sodium channels expressed in tsA201 cells. Am. J. Physiol..

[10] Yang XR, Ginjupalli VKM, Theriault O, Poulin H, Appendino JP, Au PYB (2022). SCN2A-related epilepsy of infancy with migrating focal seizures: Report of a variant with apparent gain- and loss-of-function effects. J. Neurophysiol..

[11] Richards S, Aziz N, Bale S, Bick D, Das S, Gastier-Foster J (2015). Standards and guidelines for the interpretation of sequence variants: A joint consensus recommendation of the American college of medical genetics and genomics and the association for molecular pathology. Genet. Med..

[12] Claes LR, Deprez L, Suls A, Baets J, Smets K, Van Dyck T (2009). The SCN1A variant database: a novel research and diagnostic tool. Hum. Mutat..

[13] Solé L, Tamkun MM (2020). Trafficking mechanisms underlying Na(v) channel subcellular localization in neurons. Channels (Austin, Tex).

[14] Thompson CH, Porter JC, Kahlig KM, Daniels MA, George AL (2012). Nontruncating SCN1A mutations associated with severe myoclonic epilepsy of infancy impair cell surface expression. J. Biol. Chem..

[15] Han Z, Chen C, Christiansen A, Ji S, Lin Q, Anumonwo C (2020). Antisense oligonucleotides increase Scn1a expression and reduce seizures and SUDEP incidence in a mouse model of Dravet syndrome. Sci. Transl. Med..

[16] Levy MA, McConkey H, Kerkhof J, Barat-Houari M, Bargiacchi S, Biamino E (2022). Novel diagnostic DNA methylation episignatures expand and refine the epigenetic landscapes of Mendelian disorders. HGG Adv..

