# Supplementary material for: De novo Y1460C missense variant in NaV1.1 impedes the pore region and results in epileptic encephalopathy
Source: Sci Rep. 2022 Oct 13;12:17182. doi: 10.1038/s41598-022-22208-x (PMC9561119; doi:10.1038/s41598-022-22208-x)
Supplement: Supplementary file 1 — Supplementary Information. [file 41598_2022_22208_MOESM1_ESM.docx]

**De novo Y1460C missense variant in Na_V_1.1 impedes the pore region and results in epileptic encephalopathy.**

Quentin Plumereau^1^, Aya Ebdalla^2^, Hugo Poulin^1^, Juan Pablo Appendino^2^, Morris H. Scantlebury^3^, Ping Yee Billie Au^3^, and Mohamed Chahine^1,4^*

^1^CERVO Brain Research Center, Quebec City, QC, Canada

^2^Department of Medical Genetics, Alberta Children’s Hospital Research Institute, Cumming School of Medicine, University of Calgary, Calgary, AB, Canada

^3^Department of Pediatrics, Neurology Section, Alberta Children’s Hospital, Cumming School of Medicine, University of Calgary, Calgary, AB. Canada.

^4^Department of Medicine, Faculty of Medicine, Université Laval, Quebec City, QC, Canada

**Supplementary Information**


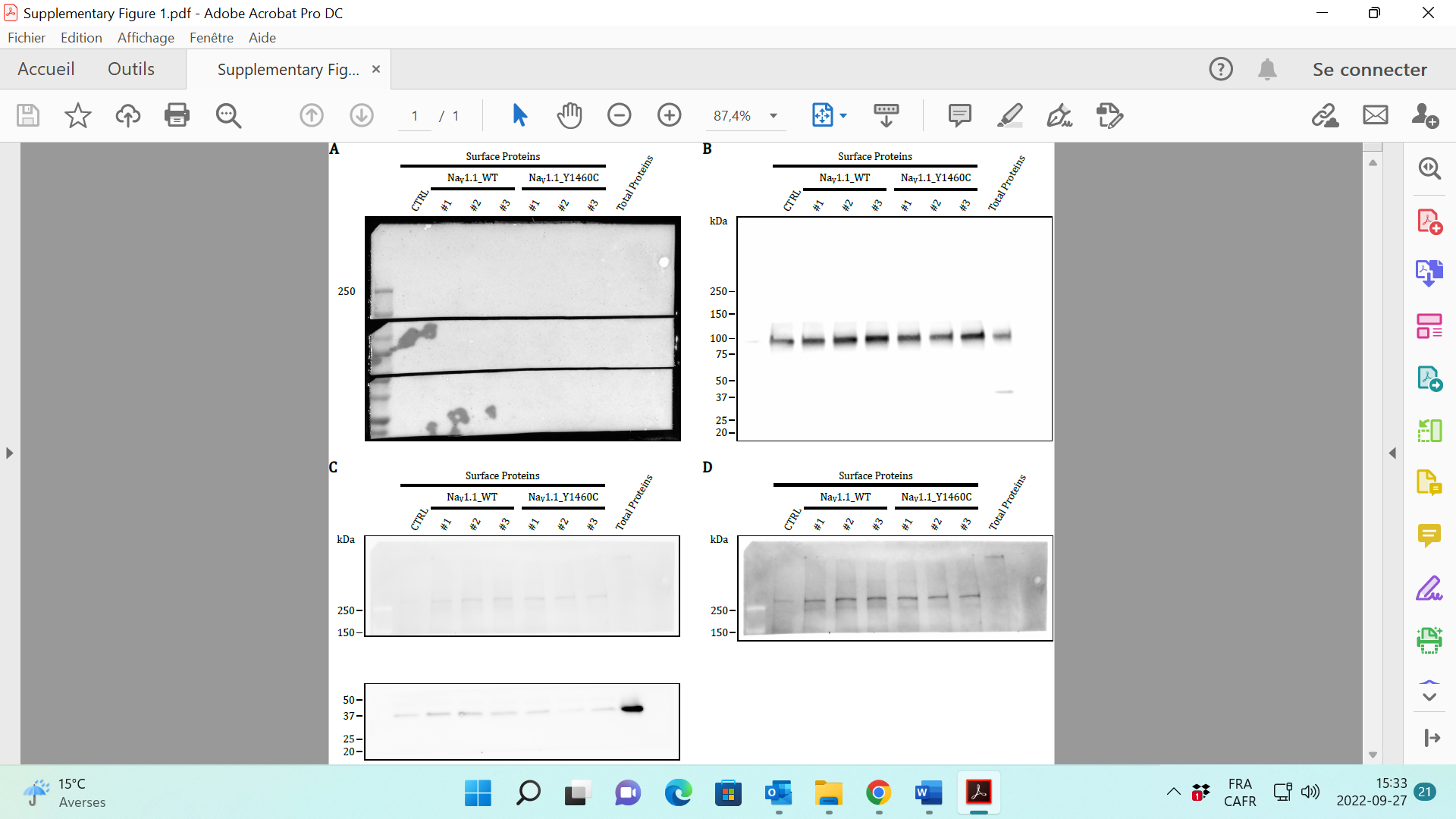


**Supplementary Figure 1: Uncropped images of biotinylated protein blots and protein standards for Figure 4.** Membrane was cut into 3 sections prior to blotting. All blots were exposed at the same time, then individually removed when near saturation to properly auto expose the next section. **A)** Colorimetric picture of the protein ladder and chemiluminescence exposure of **B)** NaKATPase **C)** Na_v_1.1(Top panel, low exposure) GAPDH (lower panel) and **D)** Na_v_1.1 (high exposure)
